# Use of biologics in pediatric-onset hidradenitis suppurativa: a case series^؆^

**DOI:** 10.1016/j.abd.2025.501188

**Published:** 2025-08-14

**Authors:** Gilberto Pires da Rosa, Sofia Magina, Carmen Lisboa, Filomena Azevedo, Alberto Mota, Maria João Cruz

**Affiliations:** a Department of Dermatology and Venereology, São João Local Health Unit, Porto, Portugal; b Department of Medicine, Faculty of Medicine, University of Porto, Porto, Portugal; c Biomedicine Department, Faculty of Medicine, University of Porto, Porto, Portugal; d Microbiology, Department of Pathology, Faculty of Medicine, University of Porto, Porto, Portugal; e CINTESIS@RISE ‒ Center for Health Technology and Services Research, Porto, Portugal

Dear Editor,

Recent literature reveals a bimodal distribution of Hidradenitis Suppurativa (HS), with the first peak occurring in late adolescence and the second in the fourth decade of life. Most publications focus on adulthood, with limited research addressing pediatric HS,^1^, ^2^ particularly concerning the use of biological agents in this population.^2^, ^3^, ^4^

Aiming to add further data to this topic, we performed a retrospective analysis of patients diagnosed with HS under the age of 18 at a Portuguese tertiary university hospital, selecting those who initiated their first biologic before adulthood.

Twelve patients were included in the analysis (8 female, 67%), with a median age at diagnosis of 16 years (range 10‒17 years). The median time from symptom onset to diagnosis was 27 months (IQR 14‒34.3-months), and the median follow-up duration was 6-years (IQR 3‒8.8-years). At diagnosis, 67% (n = 8) were classified as Hurley Stage III, 25% (n = 3) as Stage II, and 8% (n = 1) as Stage I. Additional clinical characteristics and previous therapies are available in Table 1. Regarding biologic therapy, adalimumab was administered to all 12 patients, ustekinumab to 4 patients (33%), and infliximab and secukinumab, each to 3 patients (25%). A mean of 2 biologics were administered per patient (range 1‒4). The median age for starting biologic therapy was 16.5 years, with therapy initiated before age 18 in all adalimumab patients (ages 11 to 17), in 2 out of 4 ustekinumab patients (both aged 17), and in 2 out of 3 infliximab patients (aged 13 and 17). Secukinumab therapy was initiated during adulthood. Adalimumab (n = 12) had primary failure (no response at week 16) in 17% of cases (2 patients), secondary failure in 50% (6 patients, median duration 24 months), and maintained efficacy in 33% (4 patients, median follow-up 36 months). Ustekinumab (n = 4) exhibited primary failure in 1 patient, with maintained efficacy in the remaining 3 (with a median follow-up of 23-months). Infliximab (n = 3) showed secondary failure in all 3 cases (median duration of 18 months), and secukinumab (n = 3) exhibited primary failure in 1 patient, with maintained efficacy in the remaining 2 (with a median follow-up of 12 months). Complete results are shown in Table 2. Dose intensification of adalimumab, from 40 mg to 80 mg/weekly, was attempted in 5 patients, without therapeutic benefit. Overall, 42% (n = 5) of patients on biologics achieved controlled disease – defined as improvement from severe to mild in the Hidradenitis Suppurativa Severity Score System (IHS4), while 33% (n = 4) achieved partial control – IHS4 improvement from severe to moderate, and 25% (n = 3) had uncontrolled disease (no IHS4 improvement). While on biologic, 83% of patients underwent HS surgery (n = 10), with the same percentage requiring oral antibiotics (n = 10), 33% requiring oral corticosteroids (n = 4), and 25% oral retinoids (n = 3). None of the patients submitted to surgery discontinued biologic therapy before the intervention, and all reported local improvement in their condition following the procedure. Finally, we matched our cohort with 16 pediatric-onset HS patients from our center who did not require biologic therapy, for comparison of baseline clinical characteristics. These groups displayed no significant differences regarding sex, age, and age at diagnosis. Patients in the biologic group displayed higher Hurley scores at diagnosis (p < 0.001), more perineal involvement (p = 0.03), and higher probability of undergoing surgery (p = 0.005).

**Table 1:** Clinical characteristics and previous therapies of the pediatric-onset hidradenitis suppurativa patients treated with biologics (n = 12).

Clinical data	n (%)
***Comorbidities***	
Overweight/obesity	10 (83%)
Severe acne	5 (42%)
Pilonidal sinus	4 (33%)
***Affected locations***	
Inguinal	12 (100%)
Axillary	10 (83%)
Perineal	7 (58%)
Intermammary	4 (33%)
***Previous Treatments***	
Oral antibiotics	12 (100%)
Contraceptive Pill (Female patients, n = 8)	5 (63%)
Oral retinoids	5 (42%)
Dapsone	3 (25%)

**Table 2:** Biologic therapies and treatment outcomes in the 12 pediatric-onset hidradenitis suppurativa patients.

Biologic Agent	n (%)	Age Range at Start (years)	Initiated before 18 (n)	Median duration (months)	Primary failure, n (%)	Secondary failure, n (%)	Maintained Efficacy, n (%)
**Adalimumab**	12 (100)	11‒17	12	24	2 (17%)	6 (50%)	4 (33%)
**Ustekinumab**	4 (33)	17‒24	2	21	1 (25%)	‒	3 (75%)
**Infliximab**	3 (25)	13‒23	2	18	‒	3 (100%)	‒
**Secukinumab**	3 (25)	18‒26	0	6	1 (33%)	‒	2 (67%)

The use of biologics in pediatric-onset HS remains underreported. A recent systematic review of medical treatments in pediatric HS included 33 patients treated with biologics,^4^ with 12 derived from a single study,^3^ and the remainder from case reports or small case series. Clinical improvement was reported in 94% of patients receiving biologic therapy, a higher response rate than observed in our cohort. This discrepancy may partially reflect shorter follow-up durations and potential positive publication bias in published case reports. In our cohort, a noteworthy need for additional therapies was also observed among patients on biologics, including oral antibiotics, corticosteroids, and retinoids. Despite this, our findings reinforce the efficacy and significant role of biologics, namely adalimumab, in this population, although no benefit was noted with dose escalation. Ustekinumab, though off-label, also appears to be a valid therapeutic option for adolescents. Surgery is increasingly recognized as a complementary therapeutic approach alongside medical therapy, enabling further management of HS lesions following initial improvement achieved with biologics, with positive results in our patients. Nevertheless, our data additionally reveal a striking prevalence of overweight/obesity in this pediatric population, which should be addressed, and a significant diagnostic delay, with a median of 27-months from symptom onset to diagnosis, and most patients already displaying severe disease at this time. This highlights the need for earlier recognition, facilitating timely access to these therapies, with the goal of avoiding disease progression and reducing cumulative damage.

## Research data availability

The data that support the findings of this study are available from the corresponding author upon reasonable request.

## Scientific Associate Editor

Hiram Larangeira de Almeida Jr.

## Financial support

This research received no specific grant from any funding agency in the public, commercial, or not-for-profit sectors.

## Authors' contributions

Gilberto Pires da Rosa: The study concept and design, data collection, or analysis and interpretation of data, statistical analysis, writing of the manuscript or critical review of important intellectual content, critical review of the literature, final approval of the final version of the manuscript.

Sofia Magina: The study concept and design, effective participation in the research guidance, intellectual participation in the propaedeutic and/or therapeutic conduct of the studied cases, and final approval of the final version of the manuscript.

Carmen Lisboa: The study concept and design, effective participation in the research guidance, intellectual participation in the propaedeutic and/or therapeutic conduct of the studied cases, and final approval of the final version of the manuscript.

Filomena Azevedo: The study concept and design, effective participation in the research guidance, intellectual participation in the propaedeutic and/or therapeutic conduct of the studied cases, final approval of the final version of the manuscript.

Alberto Mota: The study concept and design, effective participation in the research guidance, intellectual participation in the propaedeutic and/or therapeutic conduct of the studied cases, and final approval of the final version of the manuscript.

Maria João Cruz: The study concept and design, data collection, or analysis and interpretation of data, statistical analysis, writing of the manuscript or critical review of important intellectual content, effective participation in the research guidance, intellectual participation in the propaedeutic and/or therapeutic conduct of the studied cases, critical review of the literature, final approval of the final version of the manuscript.

## Conflicts of interest

None declared.
